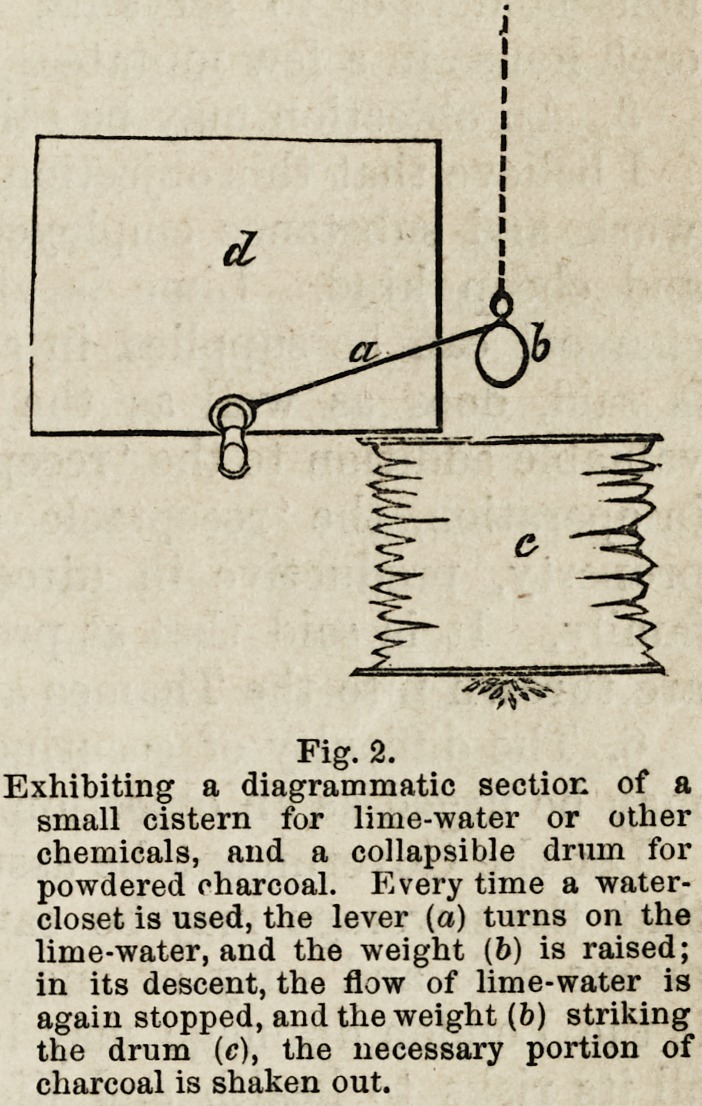# Proposal for the Drainage of London

**Published:** 1857-04

**Authors:** Thomas Hawksley

**Affiliations:** Physician to the Margaret Street Dispensary for Consumption.


### A PROPOSAL FOR THE DRAINAGE OF LONDON.

By THOMAS HAWKSLEY, M.D., L.R.C.P., Physician to the Margaret Street Dispensary for Consumption.

THE subjoined proposal to drain London by the existing system of continuous tubes or sewers, together with the addition of a detailed system of filtering, deodorising, and chemically fixing receptacles, was sent in to the new Commissioners by the author.

It is impossible to exaggerate the importance of the subject commonly called the 'London Drainage,' by which term is understood not only the removal of the surface and waste water, and of the strictly fluid refuse of the town, but also of a vast amount of its solid refuse—a refuse which, left to the present most imperfect system of removal, is found steaming up from the floors of thousands of its habitations, and from the gulley-holes of its streets; being in this way not simply revolting to the senses, but most injurious to the health. This is, however, but the beginning of evils; by the indomitable industry and perseverance of Dr. Snow, the Registrar-General, Dr. Farr, and others, evidence, amounting in strength almost to a demonstration, has been afforded, that from this source our springs and the river water can be, and are, made vehicles for conveyance of cholera poison. It is extremely probable that many other specific diseases will be found to be conveyed in the same way. Where, however, it is not the *direct* and *immediate cause* of disease, there can be no doubt that it is at all times injurious to health; and seeing how constantly the development and the force of cholera and fevers are found associated with the existence of the disgusting odours of illdrained habitations, it is a fair inference that they, the poisoned air or water, supply the required *tertium quid* without which the seed of the disease would have been unfruitful.

Of the fact that water is frequently the vehicle of disease, a striking instance occurred to the late Rev. Dr. Gilly, who, desiring, on his preferment to an elevated post in the Church, to leave behind him some token of his affection for the poor of the flock he was about to bid adieu to, took first to his counsel the medical man of the parish, who advised him neither to leave a store of port wine, nor of porter, to resuscitate the poor convalescents from ague; for the latter disease was endemic to the place, and was its constant scourge; but to build for them a deep well to supply them with pure water. Little did the good man dream of the benefits he was to be the happy means of conferring. The well was built; the water was used in preference to the shallower springs and surface water, and from that time *ague ceased in that district.* The Bishop referred to the fact in his annual charge delivered at that time.

It is natural that medical men, to whom these considerations must be matter of every day observation and thought, should enter earnestly into their study; they would be traitors to their calling and to their trust, did they not point out any adversary to the public health coming within their knowledge, and use their best abilities to promote its extermination. This must be my excuse for presenting my views on a question which may be thought to fall more correctly to the province of the engineer than of the physician.

Before describing the plan proposed to remedy the evils complained of, I beg to point out as briefly as possible some of the objections that I hold to other schemes which have been published:—

1. All these plans propose to convey both solid and fluid matters in a system of continuous tubes. I submit that such a practice is unphilosophical, opposed to the analogy of nature, and contrary to common sense; for the frequently recurring obstructions and leakages found actually to occur, might be easily predicted anterior to experience. It is difficult to conceive a justification for the adoption of such a system, except the despair of finding an alternative, after careful inquiry and experiment to discover one.

2. The adoption of a system of continuous tubes for the removal of both fluid and solid excretions, leaves the risk of atmospheric and spring water poisoning just where it now is.

3. The plans, which propose simply to extend and continue the present system of drainage, and to make the outfall of the sewerage into the Thames some miles below the metropolis, are not only open to the two great objections before mentioned, but also to that of throwing away and wasting a property in all probability valuable enough at least to pay all the expenses incurred in maintaining the town in a state of sanitary sweetness and purity, to say nothing of any ill effects that might result from the discharge of so much animal matter into the river, even at that distance. I submit that the adoption of such a plan would be the confession of great weakness, and of inability to wield the powers of mechanism and of chemistry.

4. The ingenious plans advocated by Dr. Marshall Hall and by Dr. Copland are open to the first two objections, and to those arising from the fact that the unstable chemical elements of sewerage matter are subject to rapid change and loss; and that, by the time the sewerage reached the cloacæ or the reservoirs, it would already have experienced much deterioration; then would come the difficulties of getting it into a manageable form for transmission into the country; and last, though not least, the nuisances, and the possible evils, of dealing with such large accumulations within the town.

The plan I venture to propose is one, the analogy of which I find in nature. Every animal body exhibits a sewerage work tenfold more elaborate and successful than the engineer can hope to accomplish; but nature is never found attempting to convey mixed solids and fluids in a network of continuous tubes. The only instances to the contrary are those of disease and danger, such as cases of renal and biliary calculi, detached particles of fibrin in the bloodvessels, etc. For the conveyance of the fluid excretions, a system of continuous tubes, analogous to our existing sewers, is employed, and for the sake of distinction the principle concerned in their use will be called that of 'continuity;' but, for the removal of the more solid excretions, a vast number of minute excreting receptacles, which are involutions, more or less simple, of the body's surfaces, are employed. From these the contents can be easily and conveniently cast forth, and the disorder of individual receptacles interferes not with the action of the general body. The principle concerned here is distinguished as that of 'detail.'

I propose to apply these principles to the requirements of the London sewerage, thus: By 'detail,' I would oblige every house, or in some cases blocks of houses, to be provided with a receptacle, into which the waterclosets of the house would empty themselves. The fluid and solid matters, thus received, would in the receptacle be separated into a comparatively pure water, which would filter through it, and into a solid matter which would be stored within it, and be at the same time deodorised and disinfected, and fitted for removal, in the same way as the dust of the house, at appropriate intervals and times.

By 'continuity,' the existing sewers and drains would be employed for the conveyance of the surface and waste water, together with the water filtered through the receptacles already mentioned.

The only proposal at all like the one herein sketched, is one with which I have been made acquainted since writing a letter to Sir Benjamin Hall on the subject, which appeared in the *Times* of the 3rd February. That plan is entitled "An intercepting system of sewerage, by Charles Penfold, Esq." The principal differences between the two plans consist in the absence of the filtering apparatus in Mr. Penfold's plan, in the non-employment of disinfectant and deodorising substances, in the manure being conveyed away in a fluid or semi-fluid form, also in the much greater expense and complication of his (Mr. Penfold's) plan.

The receptacle would vary in size from 1½ to 3 feet cubic capacity. It might be built of sheet iron, or of brick cemented within, so as to be waterproof, or glazed tiles might be used in its construction. At its summit, or near thereto, the house drain, conveying the solid and fluid excretions from the water-closets, together with the necessary amount of water to flush them, would enter. From the other side, near the bottom, the exit drain would connect it with the main sewer. A filtering screen of gravel would be placed in front of the opening of the latter. (Fig. 1, c.)

The filtering screen would be constructed of two large perforated slabs or tiles of fireclay, capable of sliding down and fitting in chases, left in the cement lining the receptacle. They would be arranged somewhat obliquely, as seen in the diagram, and be separated about ten inches below, at the floor of the receptacle, and about four inches above. A little coarse canvas applied to the inner side of the tiles would prevent the gravel falling through the perforations, and the interspace between the two perforated slabs, could be thoroughly packed with gravel.

The top of the receptacle would be covered in air tight by an oak or iron flap cover.

The above described apparatus constitutes the essential machinery of my plan. Its perfection would consist in erecting, in new houses, the waterclosets outside the house, in connexion with balconies and verandahs, in the greenhouse style, combining by this arrangement, convenience, salubrity, and the opportunity of communicating by a short, straight, cast iron tube, directly with the receptacle.

Fig. I.
Diagrammatic section of a 'receptacle', shewing: A. The entrance pipe or house drain. B. The exit pipe to sewer. C. The filtering screen, consisting of two large slabs or tiles of fire-clay, freely perforated, and packed in their interspaces with gravel. D. The lid or cover of receptacle.

To separate the solids of the urine, and to fix the ammonia and other gases evolved from the excretions, it is believed that caustic lime, sulphate of lime, and charcoal or soot, will prove very efficient chemicals. I propose the employment of small quantities of these, or some of these, together with cinder-dust. Portions of these it is necessary to throw into the receptacle from time to time. Deodorisation, and, there is every reason to believe, disinfection, are accomplished by the same agencies, and at the same time, as the chemical changes last referred to are produced.

EXPERIMENTS. The time which has elapsed since I took up this subject, to that in which I was compelled to send in my plan, would not permit me to appeal much to the test of experiment. I have done my best, and can only regret the meagreness of my report in this respect. A receptacle on this principle has been erected by a gentleman at a model lodging house in Chelsea; but it is scarcely completed, and there has been no time to test it.

I have experimented with a large glass receiver, fitted with a filtering screen of gravel placed between two layers of perforated zinc. The required conditions were carried out as strictly as possible, and the results quite came up to those expected.

A layer of cinder-dust was first laid down; then some soot; then a mass of human fæces; and lastly a quantity of urine, mixed with lime-water, was thrown in. The water which filtered through, though coloured by the soot, had very little odour, and that was of ammonia and soot only; and it had a much lower specific gravity than that of the mixed fluid thrown in (urine and lime-water).

In another instance, when charcoal was used as the deodoriser instead of soot, the fluid escaping was nearly colourless.

The solid manure left in the receiver was in all cases a rich black looking solid, with a strong odour of soot and ammonia, but otherwise deodorised.

My next anxiety was to assure myself of the separation of the solids of the urine, and of the best mode of accomplishing it. The most striking results were obtained by the employment of a strong lime-water, manufactured by Mr. Bastick, of Brook Street, Hanover Square, to whose skill as a scientific chemist I am indebted for some examinations of the results of my experiments. The quantity of precipitate thrown down from urine by this reagent is astonishing. Liebig has recommended sulphate of lime for this purpose; and it is believed that, in addition to throwing down the phosphates of the urine, it will fix the ammonia set free by decomposition of the urea. These points remain for investigation. The experiments already performed have been done with caustic lime and soot, or charcoal alone; but I believe that great improvements may be effected in this part of the subject, and I do not despair of seeing nearly all the solid matter of the urine thrown down and fixed by chemicals, so as to permit of its employment in agriculture, and the arrest of its loss in the sewers.

Mr. Bastick thus reports:—

"From a pint of fresh urine in a normal state, to which concentrated lime water had been added, nearly as long as that agent would produce any precipitate therefrom, a quantity of solid matter was obtained, which, after being thoroughly washed, to remove any adhering bodies soluble in distilled water, and then dried, at a temperature of 212°, weighed 53· grains. To ascertain by a simple means, which, although having no pretention to rigid scientific analysis, yet will indicate with tolerable precision the object in view—viz.: what portion of this precipitate consisted of organic matter derived from the urine—it was exposed to a red heat for half an hour, and again weighed. The loss by heat was found equal to 13, or 2 per cent., clearly showing that the lime water had removed from the urine at least 7 grains per pint of organic matter.

"The precipitate, after being heated to redness, consisted principally of the phosphoric acid of the urine, in combination with the lime previously existing in that fluid, and that which had been subsequently added. But, as in Dr. Hawksley's plan for removing the sewerage of the metropolis, and for employing such sewerage in agriculture, no washing of the matters precipitated or solidified by the lime would take place, consequently the figures above related would unfairly present the amount of solid matter removed. In order to learn the amount of this solid matter, the precipitate from a pint of urine was simply separated by filtration, and then dried at 212°. It now weighed 89 grains.

"I also examined a specimen of the fluid which had passed through Dr. Hawksley's filtering receptacle. I found in it a perfect *absence of the phosphates*, which had been solidified and retained in the receptacle with the solid excretions. In short, the fluid in question resembled in most of its qualities the urine which had been previously treated with lime.

"WM. BASTICK."

### *Reply to Objections to the New Plan Proposed.*

1. It may be said that the plan involves a return to the disgusting system of cesspools.

The answer to this is, that the 'receptacle' is wholly unlike the cesspool, both in principle and in action; it is in fact a *filter*, in which the residuum of filtration is indeed accumulated for a time, but in a form and quality quite unlike the contents of a cesspool; it is harmless to health, subject as it would be to frequent removal; it is not disgusting to the senses, and it admits of removal without inconvenience or annoyance.

2. It may be said that the inhabitants of houses would not attend to the management of the receptacles, *i.e.*, supply them with the necessary chemicals and deodorisers; and that they would not submit to anything which gave trouble.

The reply is that the 'receptacle' may be made to manage itself in the intervals of its being emptied, by attaching to it a small cistern of lime-water, mixed, if necessary, with other chemicals; and a valved drum of powdered charcoal. Both of these may, without complication, expense, or difficulty, be made to emit the required portion of their contents with every employment of a watercloset. (*Vide* fig. II.) At all events, the contractors for the manure might be made the responsible parties to attend to this feature of the plan.

Fig. 2.
Exhibiting a diagrammatic section of a small cistern for lime-water or other chemicals, and a collapsible drum for powdered charcoal. Every time a water-closet is used, the lever (*a*) turns on the lime-water, and the weight (*b*) is raised; in its descent, the flow of lime-water is again stopped, and the weight (*b*) striking the drum (*c*), the necessary portion of charcoal is shaken out.

The objection of trouble resolves itself into a balance of advantages. On which side are the greatest, on that of the *laissez faire* system, with its filth and insecurity to life, or on that of activity, with a slight temporary inconvenience followed by the blessings of pure air, water, etc.?

3. It may be objected that every house does not present suitable accommodation for a 'receptacle.'

Almost every house possesses a front or a back area, in which the instrument could be bestowed beneath the pavement; but where no areas exist, a larger kind of receptacle might be constructed beneath the footway, which would accommodate the requirements of several adjoining houses.

4. Objections may be raised against any supposed nuisance arising from the *removal* of the manure, either from any uncorrected odour, or from the time of day at which the removal might be effected. Also from any exaggerated notion of the expense of the cartage to be employed in it.

The reply is, that what little odour the manure might emit would be very conveniently removed by throwing over it a layer of dry cinder dust, and it might be found desirable to remove the contents of the 'receptacle' and of the 'dust bin' at the same time, and by the same organisation of carts. The addition of the manure would not impose above another third of labour on the existing arrangements for the removal of the dust. This replies also to the question of the expense and difficulty of cartage.

As to times for the removal, either after dark or early in morning would be productive of very little inconvenience to householders or to servants. The object would be effected for each house in a few minutes.

5. An objection may be raised to the expense of the plan.

I believe that this objection need not be a difficulty, for every work and substance employed by my scheme is of a common and cheap kind. Lime is abundant, and economical. Peat charcoal can be supplied in any quantity. Peat uncharred, it is said, does as well as the charcoal, and it might prove a valuable addition to the 'receptacle.' On the other hand, when in operation, the 'receptacle' becomes to the proprietor a little property, productive in direct proportion to the size of his family. It is said that at present £400,000 worth of manure are thrown into the Thames annually.

6. The difficulty of securing a general adoption of the plan may be urged as an objection.

Let the Government be assured, by well tested experiments, that the sewerage may be saved for agricultural purposes, by means safe and easy of adoption, and that consistently with it the London drainage may be much simplified and robbed of all its risks to health and life; then nothing more, it is presumed, will be needed, than to make it an offence against the law to run any sewerage or other solid or offensive matter into the drains, but such as had been previously purified and filtered by such a process as that explained. Companies and contractors would accomplish the rest.

## Figures and Tables

**Fig. 1. f1:**
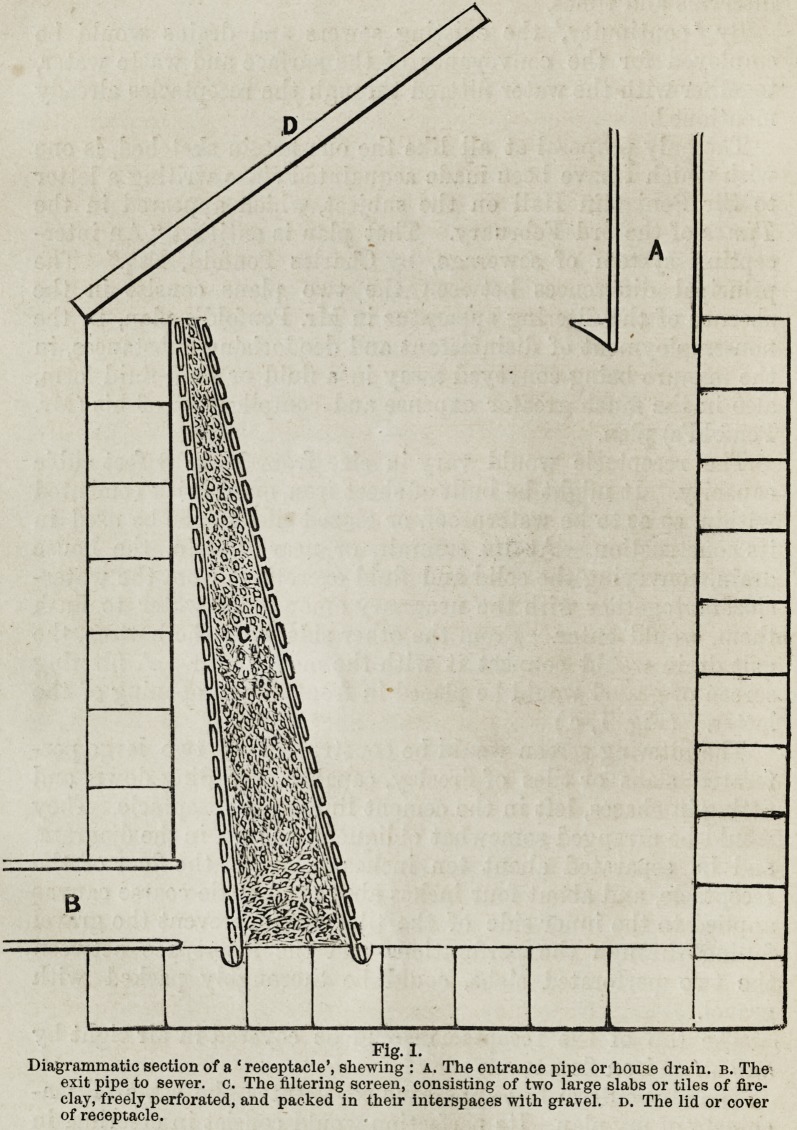


**Fig. 2. f2:**